# Supplementary figures and images for: More Than 50 Long-Term Effects of COVID-19: A Systematic Review and Meta-Analysis
Source: Res Sq. 2021 Mar 1:rs.3.rs-266574. Preprint. [Version 1] doi: 10.21203/rs.3.rs-266574/v1 (PMC7941645; doi:10.21203/rs.3.rs-266574/v1)

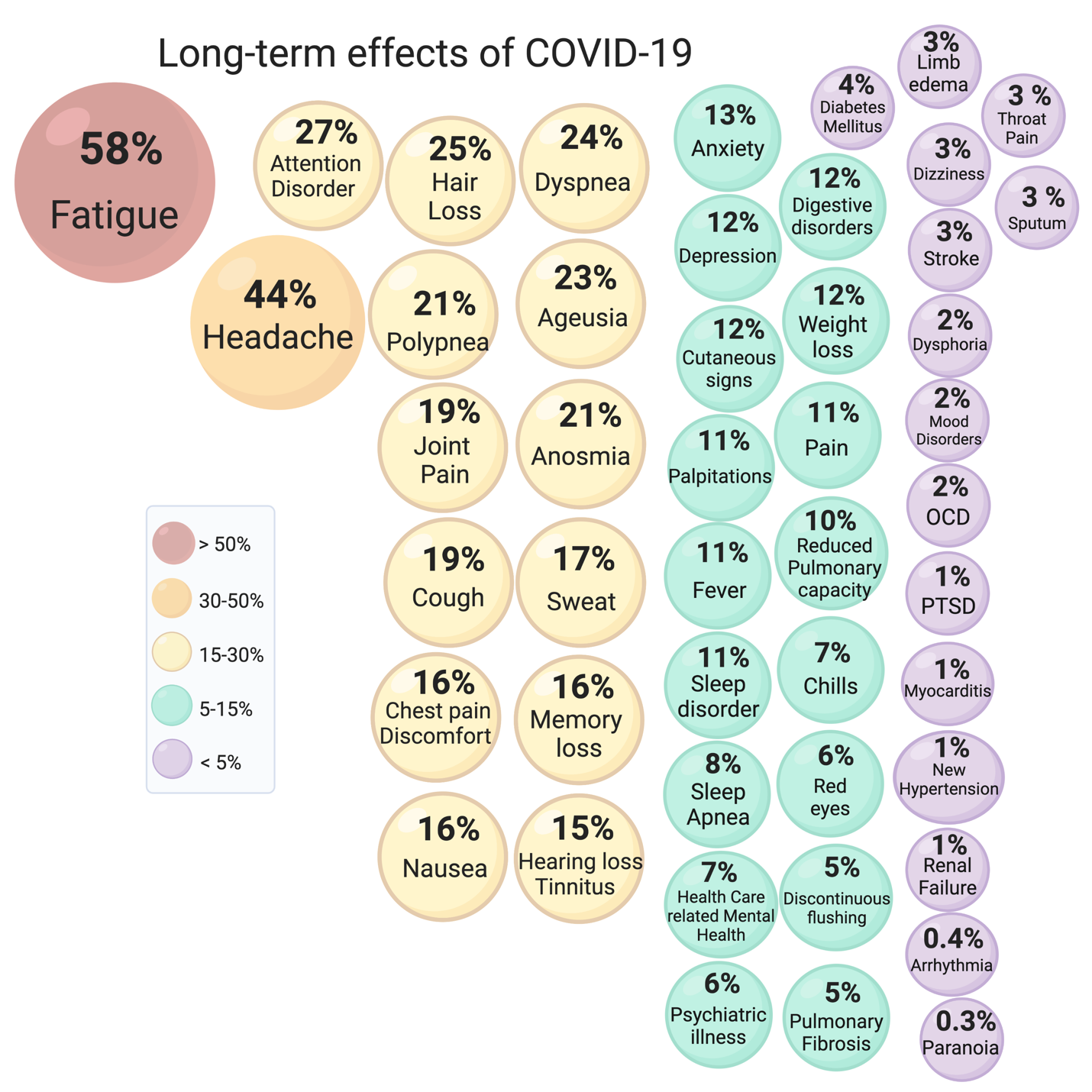

Supplement: Supplement [file 896382fb837d01f54b786d88.png]
