# Supplementary material for: More Than 50 Long-Term Effects of COVID-19: A Systematic Review and Meta-Analysis
Source: Res Sq. 2021 Mar 1:rs.3.rs-266574. Preprint. [Version 1] doi: 10.21203/rs.3.rs-266574/v1 (PMC7941645; doi:10.21203/rs.3.rs-266574/v1)
Supplement: Supplement [file 09755d2fc66887b4a6df7d1c.docx]

**Supplemental Table 1.** *Health states Quality Index variables.*

| **Study** | **1.Population and observation period well defined** | **2.Diagnostic criteria** | **3.Method of case ascertainment** | **4.Administration of measurement protocol** | **Catchment Area** | **Prevalence measure** | **Total (Max: 11)** |
| --- | --- | --- | --- | --- | --- | --- | --- |
| Andrews (40) | 1 | 1 | 2 | 3 | 1 | 2 | 10 |
| Carfi (12) | 1 | 0 | 1 | 3 | 1 | 2 | 8 |
| Carvalho-Schneider (41) | 1 | 1 | 1 | 3 | 2 | 1 | 9 |
| Chopra (42) | 1 | 0 | 2 | 1 | 2 | 2 | 8 |
| Galvan-Tejada (43) | 1 | 0 | 2 | 3 | 1 | 2 | 9 |
| Garrigues (44) | 1 | 1 | 1 | 3 | 1 | 2 | 9 |
| Horvath (45) | 0 | 1 | 2 | 3 | 1 | 2 | 9 |
| Kamal (46) | 0 | 0 | 3 | 3 | 1 | 2 | 9 |
| Mandal (47) | 1 | 1 | 2 | 3 | 2 | 2 | 11 |
| Munro (48) | 1 | 0 | 1 | 3 | 1 | 2 | 8 |
| Sonnweber (49) | 1 | 1 | 2 | 3 | 2 | 2 | 11 |
| Taquet (50) | 1 | 1 | 2 | 2 | 2 | 2 | 10 |
| Tenforde (2) | 1 | 0 | 2 | 3 | 2 | 2 | 10 |
| Townsend (3) | 1 | 1 | 1 | 3 | 1 | 2 | 9 |
| Xiong (10) | 1 | 0 | 2 | 3 | 1 | 2 | 9 |

1. 1) Yes=1, No=0
2. 2) Diagnostic system reported=1, Own system /symptoms described/no system/not specified = 0
3. 3) Community survey/multiple institutions=2, Inpatient/inpatients and outpatients/case registers=1, Not specified=0
4. 4) Administered interview=3, Systematic casenote review=2, Chart diagnosis/case records=1,Not specified=0
5. 5) Broadly representative (national or multi-site survey)=2, Small area/not representative (single community, single university)=1, Convenience sampling/ other (primary care sample/treatment group)=0
6. 6) Point prevalence (e.g. one month=2, 12-month prevalence=1, Lifetime prevalence=0
